# The experiences of foundation doctors with dyspraxia: a phenomenological study

**DOI:** 10.1007/s10459-021-10029-y

**Published:** 2021-02-09

**Authors:** Eleanor Walker, Sebastian C. K. Shaw, Malcolm Reed, John L. Anderson

**Affiliations:** 1 Frimley Park Hospital, Surrey, UK; 2 Department of Medical Education, Brighton and Sussex Medical School, University of Brighton, Falmer, Brighton, BN1 9PH UK

**Keywords:** Dyspraxia, Interpretive phenomenology, Junior doctors, Medical education, Specific learning difficulties

## Abstract

Dyspraxia, otherwise known as Developmental Coordination Disorder (DCD), is a specific learning difficulty (SpLD). Its main difficulties manifest as problems with motor coordination, organisation, academic and social difficulties. There are now more students arriving at university with SpLDs, and, therefore, a similar rise may be expected within medical education. There has been no previous research focusing on dyspraxia in doctors. An interpretive phenomenological approach was used. Six UK foundation schools disseminated the announcements. Three participants took part in loosely structured telephone interviews regarding their experiences of undertaking medical school and foundation school with dyspraxia. These were transcribed verbatim and then thematically analysed. The themes could be split into two main categories: “Weakness and Coping Strategies” and “Perspectives of Dyspraxia”. “Weakness” included: clumsiness, organisation and needing extra time. The participants focused on their “Coping Strategies” that included: Ensuring safety, adapted learning preferences and external support. “Perspectives of Dyspraxia” included: diagnosis, career choice, stigma, “normalisation” and the “difference view” or “medical deficit” view of dyspraxia. Doctors with dyspraxia often mask their difficulties through sophisticated coping strategies. These were determined and hardworking individuals who believe that their dyspraxia was a positive aspect of their identity, adopting a “difference view”. They felt further education is needed about dyspraxia to change the perceived stigma. There is now a need for further research in this area.

**Supplementary Information:**

The online version contains supplementary material available at 10.1007/s10459-021-10029-y.

## Background

**Fig. 1:**
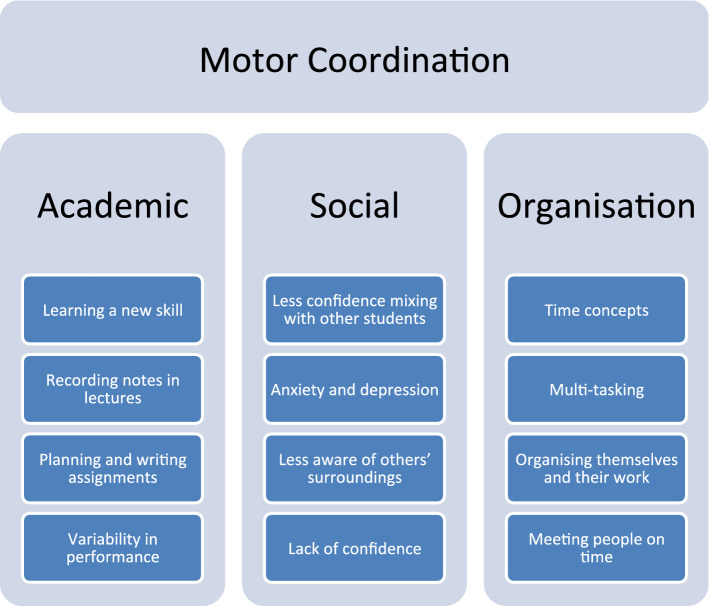
Common difficulties for individuals with dyspraxia. Adapted from the Dyspraxia Foundation (Foundation, 2012)

Dyspraxia is one of a family of Specific Learning Difficulties (SpLDs) that includes dyslexia, dyscalculia and dysgraphia (Gibbs et al., 2007). It is also known as Developmental Coordination Disorder (DCD) and historically as “Clumsy Child Syndrome” (Cantell et al., 2003; Fitzpatrick & Watkinson, 1993; Geuze and Borger, 1993; Hellgren et al., 1994; Losse et al., 1991; Missiuna et al., 2008; Skinner & Piek, 2001). Harris et al. discuss how, in accordance with DSM-5 guidelines, an individual with dyspraxia may have “motor coordination below expectations for his or her chronologic age, may have been described as “clumsy” and may have had delays in early motor milestones, such as walking and crawling” (Harris et al., 2015). Dyspraxia encompasses a wide range of features though, which extend beyond the diagnostic criteria (Cantell et al., 2003; Geuze & Borger, 1993; Losse et al., 1991; Skinner & Piek, 2001). The Dyspraxia Foundation outline a range of issues that people with dyspraxia may have difficulty with (Fig. 1) (Foundation, 2012).

Dyspraxia has an estimated prevalence of 6–10%, with 2% having severe symptoms (Gibbs et al., 2007). Its prevalence within medical education is unknown. A survey of medical schools conducted by Meeks and Herzer (2016) in the United States found that 2.7% (n = 1547) of US medical students had a disability of some kind, including “learning disabilities” (Meeks and Herzer, 2016). Dyspraxia is more common in males than females with a ratio of between 4:1 and 7:1 (Gibbs et al., 2007). This may be partially due to under-diagnosis in females due to there being less emphasis on sporting ability (Williams et al., 2015). Childhood and adolescent studies have found that dyspraxia is associated with educational underachievement, lower levels of self-worth and increased anxiety (Cantell et al., 2003; Fitzpatrick & Watkinson, 1993; Geuze & Borger, 1993; Hellgren et al., 1994; Losse et al., 1991; Skinner & Piek, 2001).

The number of students with SpLDs in Higher Education (HE) is increasing, and we might expect a similar increase within medical education (Kirby et al., 2008a, 2008b). Therefore, having an understanding of SpLDs and how they affect medical students and doctors is an important consideration for clinical educators. The General Medical Council (GMC) has placed an emphasis on supporting learners and trainees with disabilities, and ensuring that they have access to support with reasonable adjustments (General Medical Council, 2019). Medical students with a SpLD may have navigated their previous education with difficulty. The pressures of medical school may exacerbate their difficulties, prompting them to seek help and, subsequently, obtain a diagnosis at this stage in their lives (Griffin & Pollak, 2009).

Dyspraxia has high comorbidity with other SpLDs, such as dyslexia and ADHD in 35–50% of cases (Kirby et al., 2008a, 2008b). Issues with dyslexia have been better documented within medical education. A review by Locke et al. highlighted a number of support strategies that could be adopted to aid medical students with dyslexia, in accordance with the Equality Act (2010) (Great Britain, 2010). In addition, a paper by Shaw and Anderson highlighted problems faced by dyslexic doctors (Shaw & Anderson, 2018b). These included issues of bullying, feelings of isolation, and impacts on career pathway decisions within medicine (Shaw & Anderson, 2018b). These findings combined with EW’s personal experiences of dyspraxia (see ‘researcher backgrounds’) formed the drive to undertake this study.

Although many authors conflate dyslexia and dyspraxia, there is little research considering the unique experiences of those with dyspraxia in medicine (Kirby et al., 2008a, 2008b; Locke et al., 2015). This study aims to begin to fill this gap by addressing the following research question: “What are the experiences of foundation doctors with dyspraxia through medical school and foundation school?”.

### Researcher backgrounds

Best practice in the reporting of qualitative studies requires that we as authors/researchers outline our backgrounds and skillsets (Patton, 1999). “Because the researcher is the instrument in qualitative inquiry, a qualitative report must include information about the researcher. What experience, training, and perspective does the researcher bring to the field?” (Patton, 1999). This aids transparency and allows readers to confirm the credibility of the research team (Patton, 1999).

EW is a foundation doctor currently working in the UK. She was initially diagnosed with “considerable specific learning difficulties and dyspraxic tendencies” in her first year of medical school, having requested that her assessor did not use definitive terminology. Since this diagnosis, she has developed her research in this area, with the aim to support fellow medical colleagues with this diagnosis and further understand the experiences of other students and doctors.

SS is an Honorary Clinical Lecturer in the Department of Medical Education at Brighton and Sussex Medical School. He is also a junior doctor working in the United Kingdom. His teaching and research interests centre on the theme of diversity in medical education, stemming from his own experiences of studying and working with dyslexia. His philosophical beliefs are aligned with an interpretivist paradigm (see ‘Methods’), and he subsequently has a special interest in interpretive phenomenological and autoethnographic research approaches—each of which he has engaged with for multiple previous studies. He also currently teaches postgraduate workshops on qualitative interview skills and qualitative data analysis in health research.

MR is Dean of Brighton and Sussex Medical School, co-chair of the UK Medical Schools Council and chair of the MSC Education Subcommittee. A practising breast surgeon his main area of focus in education relates to assessment and associated policies concerning adjustments for candidates with dyspraxia and related conditions.

JA is a medical sociologist by training. He is a Principal Lecturer in Postgraduate Medicine at Brighton and Sussex Medical School. He has extensive experience and publications in both qualitative and quantitative methods. He has taught about qualitative methods, skills data analysis to postgraduate students or many years and has previously been the lead for research in the Department of Medical Education.

## Methods

### Philosophical underpinning

A research methodology encapsulates one’s approach to a given research question (Kothari, 2004). It allows researchers to make use of a paradigm—a set of assumptions of the world—to view the research through a “theoretical lens” (Creswell, 2013; Lavelle et al., 2013; Tavakol and Sandars, 2014a). Each paradigm has different views on the nature of reality (ontology) and knowledge (epistemology) (Cleland and Durning, 2015). Qualitative approaches assume that the world is subjective, with multiple realities, experienced differently by different individuals. The interpretivist paradigm assumes that researchers actively construct and interpret knowledge through social interactions to answer broad questions (such as ours) (Creswell, 2013; Lavelle et al., 2013; Tavakol & Sandars, 2014a). This is achieved through exploring the wider phenomena rather than trying to dissect it down to its constituent parts (Creswell, 2013; Tavakol & Sandars, 2014a). Through the process of hypothetico-inductive reasoning, the exploration of complex social phenomena may lead to a fuller understanding and new hypotheses (Tavakol & Sandars, 2014a). A qualitative approach, within an interpretivist paradigm, was therefore felt to be the most appropriate way to answer our research question—providing us with the meaning and understanding we required.

### Methodological overview

An interpretive phenomenological approach was used for this study. Phenomenology is a qualitative methodology, which explores the “lived experiences” of its participants (Tavakol & Sandars, 2014b). Phenomenology itself consists of two distinct schools—based upon conflicting philosophical beliefs (Lopez & Willis, 2004; Mackey, 2005). *Descriptive* phenomenology stems from the work of Edmund Husserl—a German philosopher (Shaw & Anderson, 2018a). This strives to access the untainted experiences of its participants, through minimizing researcher influences. This approach demands that researchers “bracket out” their existing beliefs, views and experiences so that they can approach the research with a blank canvas. It is been suggested that such studies should not even begin with detailed literature reviews, in order to minimize prior influences on the researchers (Lopez & Willis, 2004). Shaw and Anderson (2018a, 2018b) have argued that this approach may not be suitable or feasible for insider researchers (Shaw & Anderson, 2018a). *Interpretive* phenomenology, based upon the work of Martin Heidegger, requires that researchers’ prior knowledge/prejudices should be acknowledged and embraced throughout the research. These are used to attempt to uncover an underlying meaning that may not have been apparent to even the participants themselves (Lopez & Willis, 2004; Ng et al., 2013). This approach lends itself well to researchers with personal experience or involvement in an issue wishing to explore others’ lived experiences.

### Insider status

At the time of this study, EW was a medical student with dyspraxia in the United Kingdom (UK). As such, she would be considered an insider researcher in this area. Interpretive phenomenology helps us to embrace this, and to use her own experiences as a strength to guide the planning, conduct, and interpretation of the overall study (Shaw & Anderson, 2018a). EW’s reflections on her own experiences, thoughts and feelings led to the initial idea for this study and the generation of our interview topic guide (see attached supplement). Her insider status also enabled her to see links and trends within the data that may not have been so obvious to the rest of the research team. EW also kept a reflective log to record her own thoughts and feelings throughout the study.

### Research Governance and Ethics

The Brighton and Sussex Medical School (BSMS) Research Governance and Ethics Committee granted approval for this study.

### Recruitment

Six UK Foundation Schools disseminated an announcement inviting junior doctors with dyspraxia (JDWD) to participate during the Spring of 2017. Foundation schools are UK training bodies that train junior doctors for the two years between their graduation from medical school and advanced training in their chosen specialities. Foundation doctors with a diagnosis of dyspraxia by an educational psychologist or a doctor were eligible. Those with only self-reported dyspraxia were excluded. Interested JDWD were contacted and the study was explained to them. They were also sent a participant information sheet to read. Mutually agreeable times were then agreed for informed consenting and the interviews.

### Data gathering

Loosely structured telephone interviews were conducted by EW with each JDWD during Spring 2017. At the beginning of each phone call, EW again explained the aims of the study and received participants’ informed consent. The voluntary and confidential nature of their participation was stressed. The process included explicitly asking for a positive response to each section in the consent form. Only after informed consent was formally recorded did the interview begin. Each lasted for about an hour. Our interview topic guide can be found as a supplement to this paper.

### Data analysis

The interview audio-recordings were transcribed verbatim by EW, allowing for improved immersion in the data (Cohen et al., 2007). These transcripts then underwent a thematic analysis, using the approach of Cohen et al. (2007). The transcripts were first coded. This was done using pens and highlighters. Thoughts and ideas were recorded in the margins throughout this process. The generated codes were then entered into Microsoft Excel to record and review them. These codes were then compared back to the original transcripts. Particular attention was paid to codes generated towards the end of the process—ensuring they had not been missed in the earlier stages. Codes were then grouped into descriptive themes. As recommended by Cohen et al., codes and descriptive themes were then both analysed for the frequency with which they appeared in the transcripts in order to aid the identification of axial themes (Cohen et al., 2007). Concept maps were then used to visualize how the axial themes interlinked and formed the final, analytical themes. This was an iterative process involving three members of the team (EW, SS, & JA), thus providing investigator triangulation for the analysis (Patton, 1999).

## Results

Three eligible JDWD participated. All three were male doctors in foundation training. They were from three different foundation and medical schools. Table 1 provides a summary of their demographics.

**Table 1:** Summary of demographics

Pseudonym	Year of training	When diagnosed/education to date
Joe	FY1	Secondary school (diagnosed) Medical school
Nick	FY1	Secondary school (diagnosed) BSc Medical school
Andrew	FY1	Secondary school BSc Medical school MSc (diagnosed)

**Fig. 2:**
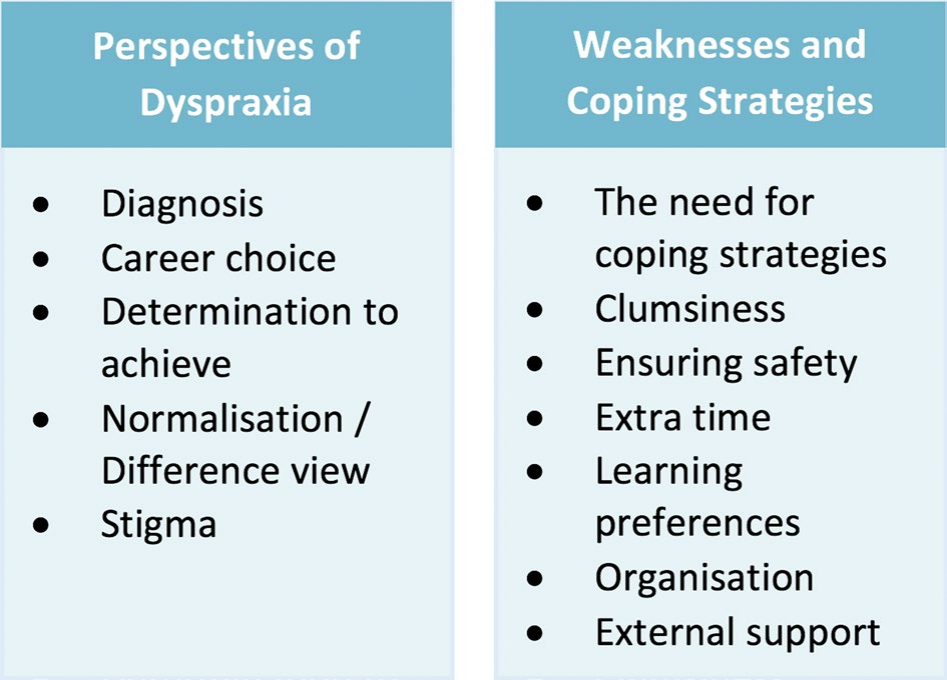
Emergent themes

The themes that emerged could be broadly split into two theme clusters (Fig. 2). Our results are subdivided accordingly.

### Perspectives of dyspraxia

#### Diagnosis

All participants reported that being diagnosed was useful as it helped explain their problems, even if it could not change them.

*“There is something really helpful to having a diagnosis… even if it doesn’t actually fix that… something I have definitely benefitted from a lot in terms of my self-confidence.” Nick*

The diagnosis also helped them to understand the coping strategies that they had already put in place and to appreciate their current performance.

*“I’ve got loads of different coping strategies that I didn’t know until I got diagnosed” Andrew*

They were able to use their diagnosis to access supports during medical school.

#### Career choice

They had all picked careers that they believed played to their strengths. Two had very detailed plans of how they were going to achieve their goals. All believed that they had the ability to undertake a surgical career path, although only Andrew had decided to pursue it.

*"I will end up going into surgery mainly because it like, it is something quite like I will get to use more than just a screen basically." Andrew*

#### Determination to achieve

All were competitive and hard-working, especially in academic areas. They believed that this was one of the key strengths resulting from their dyspraxia. All had a strong sense of the importance of ‘*proving others wrong’*. For Nick and Andrew, this was directly related to the perceived stigma surrounding their dyspraxia. This was a key motivator for them to continually improve their skills rather than becoming complacent once they became competent.

*“Almost every step of my life I have been told I couldn’t do something… then I have actually gone and ended up doing it… I am so much more resilient. I am so much more hardworking… If something is competitive, I am not really fazed by it anymore!” Andrew*

#### Normalisation/“difference view”

Each had a different interpretation of how their dyspraxia affected them. Joe recognised that he had learning difficulties but felt they did not affect his abilities.

*“I don’t see my… disabilities as disabilities, I just see them as features.” Joe*

Andrew and Nick felt their difficulties affected their daily lives, but that they were able to overcome these with coping strategies.

*“I would say nothing is disabling… but there are things that potentially like slow me down.” Andrew*

There was no consensus on which problematic areas were specific to their dyspraxia. All three had a different view as to what is considered a “normal disadvantage” experienced by everyone and what is unique to those with dyspraxia. This normalisation of certain traits meant that they did not always recognise when they had developed coping mechanisms.

*“It’s just well things that are like every day to me ends up not being normal for like everyone else, it’s just because if I didn’t, I would have difficulty with things.” Andrew*

#### Stigma

They were unanimous in their belief that there is a negative stigma in relation to dyspraxia.

*“I do feel bad about having [dyspraxia] because there is like a stigma attached to it.” Andrew*

Nick was concerned that other people would think that he was not good enough because of his differences.

*“If I know people are okay with it, I know people aren’t thinking ‘Christ, why is he such an idiot not being able to do this… if I am not worried about what people are thinking then I can focus more on actually writing things down and doing my job.” Nick*

This seemed to be the main driver for his consistent disclosure of his condition with his colleagues. He felt that if they understood his differences, they would be more accepting of them and less judgmental. He mainly reported positive experiences regarding disclosing his dyspraxia to colleagues.

All three believed that the stigma was associated with a lack of understanding about dyspraxia. Joe found the most common response to disclosing his dyspraxia was for people to ask how it affected him. He felt that, through these discussions, they were able to highlight the differences and associated strengths.

### Weaknesses and coping strategies

#### The need for coping strategies

They highlighted the importance of coping strategies to manage the challenges their dyspraxia caused. They seemed proud of their coping mechanisms, recognising them as something that positively set them apart from their peers.

*“I am definitely happy that I have got [diagnosed] and I am proud to say I have… [SpLDs] but it is only because I have these mechanisms in… spite of all the difficulties I have.” Andrew*

They also recognised that without their coping mechanisms it might have been difficult for them to succeed in medicine.

#### Clumsiness

Participants described being clumsy since childhood and having continuing issues in their adult life. They had all experienced minor incidents at work, however, felt, for the most part, that it did not affect their daily performance.

*“I was doing anaesthetics… I turned around and put a syringe on the desk, but I actually smashed the drugs tray… the whole thing went spinning across the theatre…” Joe*

#### Ensuring safety

All felt their coping mechanisms allowed them to perform safely on the wards. These mechanisms included: extra vigilance, reflection, practice and repetition, appropriate observation of challenging skills, strong verbal communication skills and avoidance of multi-tasking.

*“I am aware that I don’t do an LP every day so… asked one of the med regs… I talked him through what I am going to do first and asked if… he would come and observe me for that one.” Joe*

#### Extra time

All participants felt they needed more time to complete tasks on ward rounds, undertake clinical skills and ward tasks, and sit medical school examinations compared to their peers.

*"I got [extra time] there as well, those are the only two exams I feel like I have gotten what I deserved on." Andrew*

This caused frustration, as they felt there was a discrepancy between the ability needed in real life and that needed to pass exams.

*“If someone has the ability and the know-how and they have got that sort of like into medical school, why [would] you prevent him going any further when he has obviously got the ability.”* *Joe*

To create extra time participants described going on “pre-ward rounds” to try to get to know patients before the ward round and thus make the ward round easier for themselves.

#### Learning preferences

All displayed strong preferences for learning in ways that they felt were best suited to their difficulties. They emphasised the importance of having information in context to allow them to develop a thorough understanding of the topic.

*“I can't just stare at a screen and learn things I need to sort of understand it or talk through.”*

*Andrew*

Nick had problems learning clinical skills. He found copying a demonstration in mirror image very difficult and needed the teacher to sit next to him to allow him to follow properly.

#### Organisation

All cited what they felt to be “*disorganization”—*ranging from trouble organising themselves on a daily basis to multi-tasking whilst on the wards—as difficulties associated with their dyspraxia.

*“I feel like I am overcompensating in regards to organisation on my side but then because of it I am doing okay…” Andrew*

#### External support

All discussed the use of external support. Joe and Nick had both received extra time in exams throughout medical school. Nick had attempted to access support in the hospital. He cited a letter detailing support that he was offered, including a laptop and extra time. But Nick felt that it had had very little practical impact.

*"One of these things which become a bit laughable at this stage… the support, because it becomes a bit pretend.” Nick*

Andrew, having had no support earlier on, was still exploring the supports available to him within the hospital environment.

*"It’s just like last year I didn’t know these things were available, it's just about me going and seeing what is available now because I have found out the majority of things have made a difference for me." Andrew*

External support sometimes came in the form of supportive mentors and seniors within the clinical environment.

*"I have been really lucky to have… neurology mentors as well who have been really supportive and helped me get through a couple of projects…” Nick*

Nick and Joe found that having supportive mentors encouraged them to continue to pursue their goals as well as support their development as doctors. They found this support helped to give them confidence in their abilities.

## Discussion

The aim of this study was to examine the experiences of JDWD. The results show that they had a mixture of positive and negative experiences related to their dyspraxia—through medical school and into foundation school. Although the experience of dyspraxia was largely individual, there were some common experiences. The overwhelming determination and resilience of these individuals was inspiring. It ran throughout all three interviews. They felt that someone with more severe dyspraxic difficulties than them might be unable to survive in a clinical environment.

Their strengths, such as determination and social skills, were similar to those reported in previous studies (Griffin & Pollak, 2009; Kirby et al., 2008a, 2008b). Participants had adapted their learning preferences around their strengths. This had not previously been reported in relation to dyspraxia. They expressed the need to fully understand information to be able to retain it; developing coping strategies such as practice and repetition; and the use of a “structure” to learn information. Learning would often occur in their spare time rather than during teaching—thus affording the extra time needed.

**Fig. 3:**
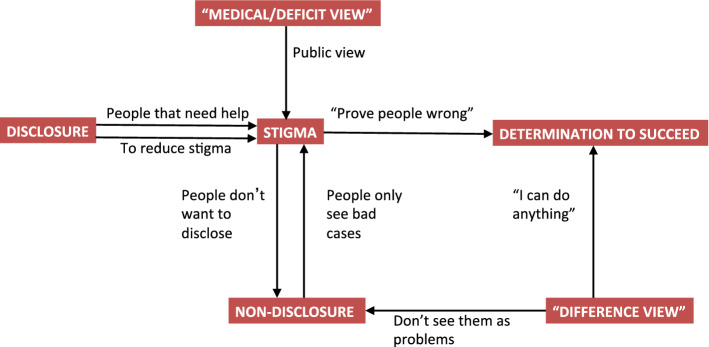
Relationship between Stigma, Disclosure, Medical/ Deficit View and Difference View

Nick discussed the relationship between disclosure and public education (see Fig. 3). He felt the public tended to see the “problem cases” because individuals with dyspraxia are more likely to disclose when they need support. Therefore, he felt others assume that *all* those with dyspraxia have problems. This may contribute to the stigma that all the participants felt surrounds dyspraxia. Hence Nick’s belief that individuals with dyspraxia should always disclose to others.

Andrew and Joe would only disclose when they felt it was relevant. They also reported ongoing concern about the stigma coming from their colleagues. Musto (2013) found that this sense of stigma led to concerns that disclosure would have a negative impact on her participants’ careers—one participant actively avoided disclosing, due to concerns of discrimination (Musto, 2013). On a positive note, Andrew was becoming more confident in disclosing his dyspraxia, especially where it enabled him to access supports. Joe also reported a generally positive response to disclosure. This contrasted with Musto, who found that many of her participants recalled negative experiences of disclosing their SpLD, which dissuaded them from doing so again. All of our participants felt that educating the public and other doctors about dyspraxia would be a positive step in reducing stigma and could make disclosure easier.

The finding that our participants had positive experiences of disclosure is important. Musto found that, if participants felt able to disclose, it allowed them to create further coping strategies within the hospital, such as asking colleagues for further support when needed (Musto, 2013).

Our participants’ experiences can be further explored using the medical and social models of disability. These can be defined thus:

“Within the medical model of disability, a disability is seen as a problem with the individual in question… emphasis is placed on “fixing” the individual… Within the social model of disability, it is believed that a disability stems from issues with the attitudes of society, causing environmental, organisational and social barriers, which act to “disable” an individual” (Walker & Shaw, 2018).

**Fig. 4:**
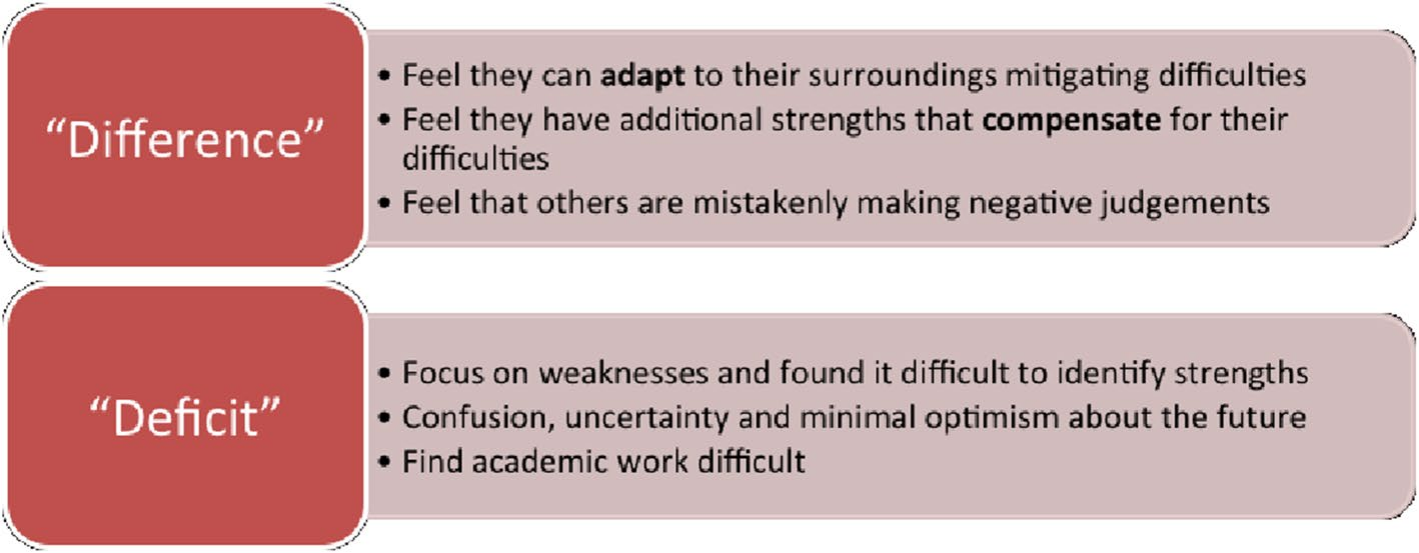
"Medical/ Deficit view" and the "Difference View" Adapted from: Griffen and Pollak^[15]^

Griffin and Pollak (2009) propose a “*medical/deficit view*” (medical model) and a “*difference view*” (social model) of SpLDs (Fig. 4) (Griffin & Pollak, 2009). All our participants fell into the “difference view” (social model), as they all had considerable career ambitions and positive clear goals. Contrastingly people that had not overcome these difficulties may not have believed they could complete medical school and may be more likely to still have a “medical/ deficit view.”

Joe believed that his dyspraxia did not disadvantage him—he believed it had helped him develop strengths. He felt that others, who have no experience of disability themselves, mistakenly make negative judgments. Nick and Andrew accepted that they had difficulty, but also believed that there were associated benefits. After a period of adaptation to their difficulties (development of coping strategies) they considered themselves to live similarly to those who are not “disabled.” All three of these are in keeping with the social model of disability.

It is important to note that these attributions are not permanent, and it is possible to change over time. This is likely to have happened to all the participants in this study with them progressing from a “medical/deficit view” to a “difference view”. Missiuna et al. found that, as her participants entered adulthood, they underwent a “reframing of the diagnosis” and became more comfortable with their condition (Missiuna et al., 2008). Our findings echo this. Andrew and Nick found that, as they became more accepting of their condition, they felt that they had more control and influence over their surroundings.

### Study strengths

This study has provided rich data about the experiences of UK foundation doctors with dyspraxia. It adds to the existing literature concerning SpLDs in medical education and provides some understanding of the experiences of foundation doctors with dyspraxia.

### Study limitations

There are a number of limitations to this study. Patton states that *“by their nature, qualitative findings are highly context and case dependent”* (Patton, 1999). For these reasons, plus the inherently small sample sizes, qualitative results are not considered generalizable in the traditional sense. It is therefore important that readers understand our results reflect the experiences of our participants and should not be taken as the experiences of *all* foundation doctors with dyspraxia in all medical and foundation schools. However, as with all qualitative studies, the results of this study should be considered transferable. We can take important lessons from these and apply them to others. Furthermore, the participants that came forward had something to say or experiences to share. Those who were struggling, or indeed those who may have felt indifferent about their dyspraxia, may have been less likely to come forward. Finally, all of our participants had comorbid specific learning difficulties. Despite this, they always signposted which condition they were talking about, enabling us to identify issues that our participants’ felt were specific to dyspraxia. However, it is worth bearing in mind that their views on which SpLD caused which experiences were self-reported.

### Considerations for further research

There are further areas in need of exploration. Research is needed to determine the prevalence of dyspraxia in UK medical schools. There is a need to explore whether the experiences reported here are specific to JDWD or are typical of junior doctors in general. There is also a need to know if such findings are shared by other JDWD in the UK. We need to develop a validated questionnaire, which could be used to expand our evidence-base. Additionally, nothing is known of the knowledge, attitudes and beliefs of doctors regarding their colleagues with dyspraxia.

There is also a need to explore what supportive adjustments are currently offered to JDWD across different foundation schools, and to consider which supports may be the most appropriate. In our study, Nick felt that his support was *“laughable”* and impossible to be practically implemented. Andrew, although actively seeking support, was unsure about what could be practically offered. Musto (2013) also found the process of seeking support for SpLDs was lengthy and, once completed, the support was often impractical due to the ward environment (Musto, 2013). In contrast, several of her participants found aspects of the support useful, especially “Dragon Medical” software to help dictate letters (Musto, 2013).

## Conclusions

JDWD report both strengths and weaknesses related to their dyspraxia. The signs of dyspraxia are often masked through overcompensation and sophisticated, practical coping strategies that enable them to function on the wards. The JDWD we interviewed were determined, hard-working, wanting to “prove others wrong”, and to excel in competitive specialities. They felt that supportive mentors and seniors who encouraged them to use their coping mechanisms were also helpful supports. There is still a perceived stigma that surrounds dyspraxia. They believed this needed to be changed through education about the reality of having dyspraxia. There is scope for further research here.

## Supplementary Information

Below is the link to the electronic supplementary material.

Supplementary file1 (DOCX 18 kb)
