# Supplementary material for: The experiences of foundation doctors with dyspraxia: a phenomenological study
Source: Adv Health Sci Educ Theory Pract. 2021 Feb 9;26(3):959–74. doi: 10.1007/s10459-021-10029-y (PMC8338860; doi:10.1007/s10459-021-10029-y)
Supplement: Supplementary file 1 — Supplementary file1 (DOCX 18 kb) [file 10459_2021_10029_MOESM1_ESM.docx]

# Interview Topic Guide

| **Topic** | **Justification** |
| --- | --- |
| Diagnosis | People may have had different experiences based on when they were diagnosed with their condition. If they were diagnosed because they were struggling they may have a more negative view of their condition than someone who was picked up on screening. Rowlands et al. found that depending on how an individual ‘framed’ their condition they had different views of their condition.^1^ |
| Life before Medical School | Students with severe dyspraxia are more likely to have had interventions before they came to medical school. Some doctors with a diagnosis before medical school may have had doubts about whether they could undertake a medical degree.  If they had struggled before medical school then they might be used to having to work hard, but others who started to struggle whilst at university may have had more trouble adapting. |
| Experiences at Medical School | The experience at medical school will be the main focus. This will be where they are developing their new skills and therefore dyspraxic students may find it harder to learn new practical skills. |
| Current Job | See above |
| Coping Strategies | If they are finding certain skills harder then they will begin to start to develop coping strategies to help make these skills easier to do. These coping strategies may vary from person to person and will also depend on what the particular problem that they are trying to encounter is. |
| Support | This is interesting as many people with dyslexia get given support and not all of them use that support to the best of its use. It would be interesting to see what support is currently being given to dyspraxic doctors, and what support they find useful. |
| Disclosure | On speaking to leading members of 2 Foundation Schools, both have said that they do not get many people with dyspraxia in their Foundation Schools. We wonder if this may reflect an issue with disclosure. |
| Career | I.e. may dyspraxic doctors choose to avoid surgery? |
| If they have both dyslexia and dyspraxia: “Which affects you more?” | This would be for just participants with both conditions. It may be that they feel that the conditions affect them more at different times i.e. lectures dyslexia is worse and dyspraxia is worse when on the wards. |

**Topics:**

1. **Diagnosis**
2. **Pre-Medical School**
3. **Medical School**
   1. **Placement**
4. **Employment**
5. **Coping Strategies**
6. **Support**
7. **Disclosure**
8. **Career Choice**
9. **(If they have both dyslexia and dyspraxia: “Which affects you more?”)**

1. Rowlands A, Abbott S, Bevere G, Roberts CM. Medical students’ perceptions and understanding of their specific learning difficulties. Int j med educ. 42013; p.200-6.
